# Effectiveness of Antimicrobial Agents Incorporated into Soft Denture Liners: A Systematic Review

**DOI:** 10.3390/ma18081764

**Published:** 2025-04-11

**Authors:** Olga Naka, Theodoros Tasopoulos, Frixos Frixou, Eirini Katmerou, Heidar Shahin, Emmanouil-George Tzanakakis, Panagiotis Zoidis

**Affiliations:** 1 Department of Prosthodontics, School of Dentistry, Aristotle University of Thessaloniki, 541 24 Thessaloniki, Greece; naka@dent.auth.gr (O.N.); eirinikatmerou@gmail.com (E.K.); 2 Private Practice, 155 61 Athens, Greece; tasopoulost@gmail.com (T.T.); frixosf08@gmail.com (F.F.); tzanakak@dent.uoa.gr (E.-G.T.); 3 Division of Prosthodontics, University of Florida College of Dentistry, Gainesville, FL 32610, USA; hshahin@dental.ufl.edu

**Keywords:** antimicrobial agent, soft denture liner, *C. albicans*, nystatin

## Abstract

Integrating soft components into denture design may significantly enhance the comfort of edentulous patients. Microorganisms, particularly *Candida albicans*, often colonize soft denture lining materials, which can release metabolic and toxic byproducts linked to the development of Denture-Induced Stomatitis. This study aimed to evaluate the effectiveness of antimicrobial agents incorporated into soft denture liners in inhibiting the adhesion and colonization of *C. albicans*. A systematic review was conducted through MEDLINE-Pubmed, EMBASE, and the Cochrane Central Register of Controlled Trials. A range of keywords was employed without applying a time filter to identify relevant literature. The review revealed many studies investigating various antimicrobial compounds added to different soft denture liner materials, all demonstrating the ability to inhibit the proliferation of *C. albicans*. All the antimicrobial agents examined exhibited a significant antifungal effect, with minimal to negligible impact on the physical properties of the denture liners. However, it was noted that the mechanical properties of the liners were modified in direct correlation to the concentration of the antimicrobial agents utilized. The successful incorporation of these agents into various soft denture liners has been documented, with nystatin being the primary pharmacological agent identified across multiple studies. While incorporating antibacterial agents was deemed successful, it is essential to note that the methodologies employed yielded varying effects on the overall performance of the soft-liner materials.

## 1. Introduction

Soft denture lining materials (SDLMs) play a critical role in the biomechanical interaction between dentures and the underlying tissues, facilitating a uniform distribution of forces during function. Integrating soft materials beneath the denture base significantly enhances patient comfort [1,2,3,4]. Furthermore, SDLMs often therapeutically retrain inflamed tissues resulting from poorly fitting dentures. Nonetheless, a notable limitation of soft denture liners is their tendency to rapidly degrade, which contributes to increased surface roughness and heightened susceptibility to microbial colonization. Research has shown that various bacteria and fungi adhere to and accumulate on the surfaces of SDLMs [5,6]. The oral environment affects the properties of soft liners. Components such as phthalates and alcohol may leach out, leading to material hardening over time and a subsequent loss of the cushioning effect. This degradation adversely affects the viscoelastic properties and creates porosities that can be filled with moisture, providing an environment conducive to colonizing, particularly *Candida* species [7]. The surface characteristics of SDLMs, salivary composition, lowered pH, and carbohydrate-rich diets contribute to the enhanced colonization and adhesion of *C. albicans* [8]. This microorganism is recognized as an opportunistic pathogen, constituting 40–80% of the microbial flora in healthy individuals and being detected in 50–98% of all patients [8,9,10,11,12]. *C. albicans* has the potential to cause tissue irritation through the release of metabolic and toxic byproducts, which may result in denture-induced stomatitis (DIS) [9].

SDLMs are particularly vulnerable to microbial contamination within the oral cavity, which complicates effective cleaning and hygiene practices [13]. A substantial body of literature has documented the ability of various fungal and bacterial species to penetrate the porous structures of denture liners, potentially compromising the longevity and structural integrity of these materials in the oral environment [14,15]. Specific cleansing techniques, while intended to maintain hygiene, may inadvertently damage soft denture liners and contribute to the exacerbation of microbial plaque accumulation [16,17]. Topical application of antifungal agents may be employed in localized infection sites, or systemic administration may be considered in more severe cases [9]. Nevertheless, the efficacy of such treatments is heavily contingent upon patient adherence to the prescribed regimen. Additionally, chemical disinfectants utilized for denture cleaning—including chlorhexidine gluconate, sodium hypochlorite, and hydrogen peroxide—have been shown to induce adverse modifications to both the chemical and physical properties of SDLMs [18,19]. Studies have indicated that integrating various therapeutic agents, such as antifungal compounds, metal oxides, and herbal remedies, into soft denture liners represents a promising avenue for mitigating DIS and enhancing the therapeutic efficacy of these liners. This integration not only prolongs the action of pharmacological agents but also aids in the treatment of associated tissue trauma [20,21]. The development of antimicrobial soft liners offers a significant benefit for patients who may be unable to perform routine denture care. While numerous antimicrobial agents have been incorporated into soft liners, a crucial question remains regarding the comparative effectiveness of these agents in inhibiting microbial proliferation and promoting optimal health outcomes for patients while preserving the desirable properties of the soft liners.

SDLMs possess notable antibiofilm properties, effectively reducing the risk of infection by resident oral microorganisms [22]. Consequently, the development of biostable dentures endowed with antimicrobial characteristics is of paramount importance. Nevertheless, despite considerable advancements in material science, biostable materials exhibiting antimicrobial properties remain elusive [23].

This systematic review aimed to critically evaluate the efficacy of various antimicrobial agents incorporated into soft denture liners in inhibiting the adhesion/colonization of *C. albicans.*

## 2. Materials and Methods

This systematic review was conducted in accordance with the guidelines outlined in the Preferred Reporting Items for Systematic Reviews and Meta Analyses (PRISMA) Statement [24]. The research question was formulated utilizing the PICOS model, specifically addressing: “Could antimicrobial agents prevent or/and treat Denture Induced Stomatitis when incorporated into soft denture liners?” In this framework, “P” denotes the population (i.e., Patients suffering from Denture-Induced Stomatitis), “I” signifies the intervention group (i.e., the application of antimicrobial agents within soft denture liners), “C” represents the comparison group (i.e., the absence of antimicrobial agents or standard treatment), and “O” outlines the desired outcomes (i.e., prevention and treatment of the condition).

A predetermined protocol strategy was implemented in accordance with PRISMA guidelines. The eligibility criteria encompassed randomized clinical trials, controlled clinical trials, and observational studies published in peer-reviewed journals. Included studies were required to have retrievable full texts and must have been published in English. Exclusions were applied to case reports, expert opinion pieces, narrative or systematic reviews, non-human studies, and studies that did not specify the percentage of incorporated antimicrobial agents. Additionally, articles addressing the release of agents without discussing effectiveness, those that solely examined the effectiveness of antimicrobial agents, studies focused exclusively on hard denture liners, articles dealing with *C. albicans* colonization or adhesion, and Letters to editors were also excluded.

Comprehensive electronic and manual literature searches were performed across databases including MEDLINE-PubMed, EMBASE, and the Cochrane Central Register of Controlled Trials, without time restrictions. The last search took place on 22 May 2023. Furthermore, references from included studies were hand-searched for additional potentially relevant research. A combination of keywords and MeSH terms were employed in the searches: (“denture liner” OR “denture lining material” OR “soft denture liner” AND “antibacterial agent” OR “anti-infective agent” OR “antimicrobial agent” OR “*Candida albicans*”). Titles and abstracts of all electronic database citations were independently assessed by two reviewers to determine compliance with the inclusion criteria, leading to the exclusion of any studies that did not meet the predetermined criteria. Data were systematically categorized based on research details, specific characteristics of the interventions and comparators, and relevant outcome measures. The quality of the included studies was evaluated by assessing the risk of bias using the ROBINS-I (Risk of Bias in Non-randomized Studies) tool [25] and the ROB2 (Risk of Bias in Randomized Trials) tool [26].

## 3. Results

**Figure 1:**
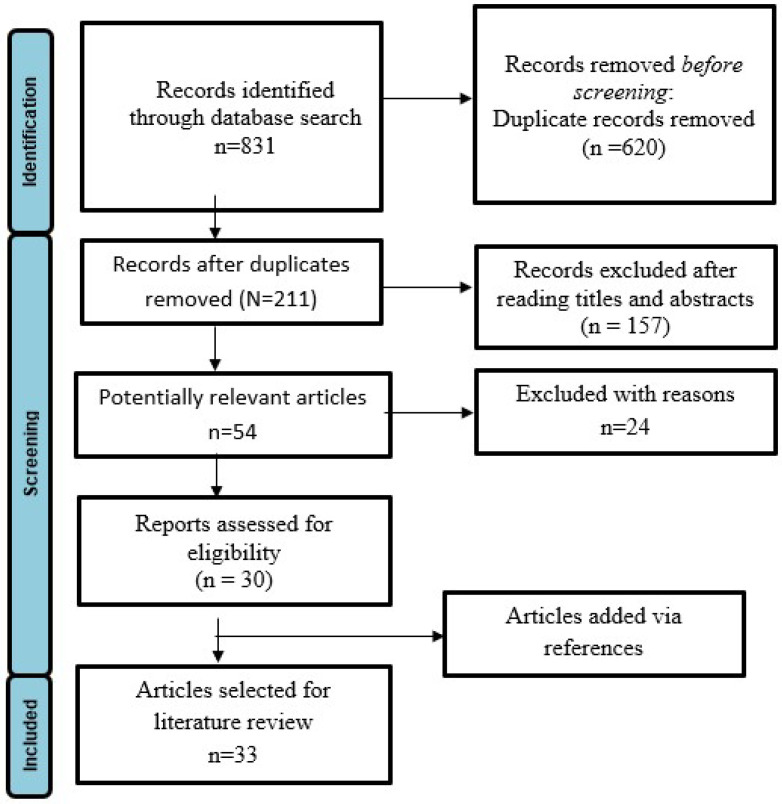
Flowchart illustrating the process of selecting studies for inclusion.

The study selection process, conducted in alignment with the principles outlined by PRISMA, is illustrated in Figure 1. After removing duplicate entries, a total of 211 publications underwent initial screening. Following a thorough examination of titles and abstracts, 54 articles were identified for comprehensive full-text review. Of these, 24 articles were excluded based on predefined inclusion criteria, leaving 30 articles deemed appropriate for further analysis. Additionally, 3 articles were incorporated through a review of references, resulting in a total of 33 publications [1,7,8,9,12,23,27,28,29,30,31,32,33,34,35,36,37,38,39,40,41,42,43,44,45,46,47,48,49,50,51,52,53] selected for qualitative analysis within the framework of this systematic review.

**Figure 2:**
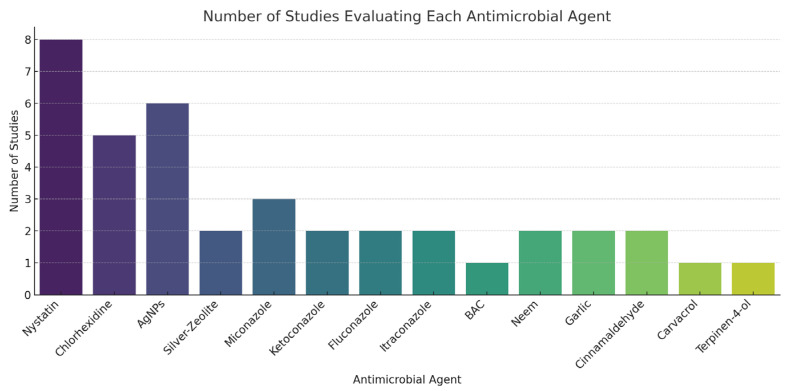
Number of Studies per Antimicrobial Agent Evaluated in the Literature.

The characteristics of the studies included in this review are summarized in Table 1 and Table 2. Out of the 33 studies incorporated into the analysis, three were conducted in vivo, while 29 were performed in vitro, and one study encompassed both methodologies. The total sample size across all the selected studies comprised 3732 patients. The studies employed a variety of antimicrobial agents, which were integrated into different types of soft denture liners, specifically Tissue Conditioners (TC), Acrylic-based (AB), and Silicon-based (SB) materials. In order to provide a consolidated view of the evidence, we summarized key findings related to each antimicrobial agent in a comparative figure. This includes the number of supporting studies, effective concentration ranges, types of soft denture liners (SDLMs) used, and reported impacts on mechanical properties. A corresponding bar chart is presented in Figure 2 to highlight the most frequently evaluated agents.

Table 3 and Table 4 present the assessment of risk of bias utilizing the ROBINS and ROB2 tools, respectively. Notably, all the included studies were found to be of high quality and exhibit low risks of bias.

A synthesized evidence table (Table 5) was created to facilitate a comparative overview of the most commonly studied antimicrobial agents. This table highlights the number of studies per agent, effective concentration ranges, the nature of the study design (in vitro or in vivo), and reported impacts on soft denture liner materials’ physical and mechanical properties. Including this comparative analysis provides a clearer understanding of each agent’s relative efficacy and material compatibility.

Nystatin emerged as the predominant antimicrobial agent, utilized in eight distinct studies [32,33,34,36,48,49,52] and in the form of nystatin alginate nanoparticles [41]. It exhibited complete inhibition of *C. albicans* at concentrations such as 1,000,000 U [52], 500,000 U [49], 1.0 mL [33], and 1–10% [36]; however, its efficacy was diminished within acrylic- based SDLMs (99.82% inhibition rate) [32]. Lower concentrations demonstrated only partial antifungicidal activity [32,33,34,36,49,52]. Notably, nystatin-loaded alginate NPs (2 mg) in TC exhibited high effectiveness [41]. Furthermore, its incorporation into antimicrobial SDLMs resulted in marked clinical improvement in cases of denture stomatitis [48]. Amphotericin B, administered at concentrations of 10 mg and 20 mg, showed limited inhibition [49,52]. In contrast, miconazole completely inhibited *C. albicans* at 250 mg in TC [49] and displayed robust antifungal activity in AB SDLM at concentrations ranging from 0.128 to 0.256 g/mL, attaining up to 99.21% inhibition, while lower concentrations (0.016–0.064 g/mL) remained effective (33.02–97.44%) [32]. Ketoconazole similarly demonstrated substantial antifungal potency, with complete inhibition at 200 mg in TC [49] and efficacy levels ranging from 93.13% to 99.29% in AB SDLM at a concentration of 0.128 g/mL; lower doses exhibited 25.39–92.69% inhibition [32].

Silver, in various formulations including silver-zeolite [45], silver-zinc zeolite NPs [37], silver NPs [1,12,40,46] and silver vanadate [42], exhibited significant antifungal properties. Silver-zeolite (5%) in TC [47] and silver NPs (2–3%) in TC [46] achieved complete inhibition, while reduced concentrations of silver NPs in silicon-based SDLMs (200 ppm) provided moderate antifungal protection (52.5%) [1]. Silver NPs in AB SDLM at concentrations of 0.2–0.3% also exhibited effectiveness, registering inhibition rates between 63.38% and 75.51% [12]. Fluconazole provided complete inhibition at a concentration of 10% in TC and exhibited strong antifungal activity at lower dosages [36]; however, a 5% concentration was recommended for short-term applications [37]. Itraconazole utilized in both AB SDLM and TC at concentrations in the range of 0.032–0.256 g/mL maintained efficacy over a period of 14 days, achieving maximum antifungal activity on day 7 for AB SDLM (97.36%) and day 2 for TC (96.44%) [32].

Various essential oils including *Melaleuca arternifolia* [53], undecylenic acid [39], origanum oil [51], *C. anthelminticum* [8], *L. usitatissimum* [8], and *Litsea Cubeba* [50] demonstrated antifungal activity at specific concentrations, with origanum oil and *M. arternifolia* achieving the highest efficacy at concentrations of 60–65% [51] and 40% [53], respectively. Carvacrol when incorporated into a SB SDLM presented an antifungal effect reaching 98%, with a concentration of 10 μL identified as most effective [30]. Chitosan, quaternized chitosan salt [44], garlic, and neem [43] also exhibited notable antifungal effects. Benzalkonium Chloride (BAC) in silicon- and acrylic-based SDLMs completely inhibited *C. albicans* at concentrations ranging from 0.5 to 5 wt% [9]. Cinnamaldehyde and Terpinen-4-ol exhibited potent inhibition, though with diminishing effects over time [35]. Cnidium officinale was effective in TC at concentrations of 400–600 μg/mL [23], while copper oxide NPs in AB SDLM (0.5–500 μg/mL) displayed dose-dependent efficacy, reaching 75% inhibition rate at the highest concentration [29]. Chlorexidine, whether as diacetate (CDC) or hydrochloride, showed compelling inhibition against *C. albicans* when incorporated into TC or AB SDLM; notably, CDC showed inhibition exceeding 99% when incorporated into AB SDLM [28,31,32,48]. Lastly, titanium oxide NPs in AB SDLM (1.0–2.0 wt%) achieved antifungal efficacy in the range of 91.42–99.28% [27].

The incorporation of various antimicrobial agents at specific concentrations did not appear to significantly influence the mechanical properties of soft liners. Notably, agents such as nystatin, chlorhexidine, ketoconazole, benzalkonium chloride, *C. anthelminticum*, *O. sanctum*, neem, garlic, and silver nanoparticles exhibited no detrimental effects on parameters including roughness, hardness, wettability, ultimate tensile strength, bond strength, tear, and flexural strength. Moreover, the addition of AgVo3 was observed to enhance adhesion between liner and the denture base material. Conversely, the antimicrobial agents terpinem4ol cinnamaldehyde, itraconazole, and miconazole were found to increase the Shore hardness and roughness of the materials [1,12,29,37,40].

According to several studies, the incorporation of antimicrobial agents into SDLM was shown to affect their physicomechanical properties, though findings remain inconsistent across the literature. Some studies reported no significant changes in these properties following the addition of antimicrobial agents, while others indicated adverse effects on particular mechanical attributes. Specifically, nystatin had no effect on the tensile strength, hardness, water sorption, or solubility of soft denture liners [35,41,52]. In contrast, the incorporation of chlorhexidine resulted in a reduction in the peel bond strength among these liners [28]. The use of silver nanoparticles demonstrated enhancements in both hardness and water sorption characteristics of pliable denture liners [1]. The addition of neem and garlic reduced the adherence of *C. albicans* without significantly affecting the hardness of soft denture liners. At the same time, cinnamaldehyde was noted for its potential to augment the rigidity of soft denture liners [35,43].

Given the inherent variability and heterogeneity of the findings across the individual studies, synthesizing the results to conduct a meta-analysis was not feasible.

## 4. Discussion

This research aims to elucidate the role of antifungal agents in inhibiting microbial growth within soft denture liners. The phenomenon of immunosuppression can precipitate the proliferation of various microorganisms, culminating in opportunistic infections commonly identified as candidiasis or candidosis, predominantly manifesting in the oral cavity [54]. A notable example is denture stomatitis, which is a prevalent form of oral candidiasis characterized by mucosal irritation beneath complete or partial removable prostheses [55].

An extensive review of the literature reveals a considerable volume of studies encompassing numerous publications and experimental investigations on antifungal pharmacological agents targeting *C. albicans*. The findings underscore the potent antifungal efficacy of these agents when incorporated into soft liner materials, demonstrating minimal to negligible adverse effects on their physical properties. Nonetheless, it was observed that the mechanical characteristics of the materials exhibited proportional alterations relative to the concentration of the antifungal agents employed. Interestingly, a variety of antimicrobial agents, when utilized at specific concentrations, did not significantly impair the mechanical integrity of the soft liners. Notably, agents such as nystatin, chlorhexidine, ketoconazole, benzalkonium chloride, *C. anthelminticum*, *O. sanctum*, neem, garlic, and silver nanoparticles exhibited no detrimental effects on properties including roughness, hardness, wettability, ultimate tensile strength, bond strength, tear resistance, and flexural strength. Furthermore, the incorporation of AgVo_3_ was found to enhance the adhesion between the liner and denture base materials. In contrast, compounds such as terpinen-4-ol, cinnamaldehyde, itraconazole, and miconazole were observed to increase the Shore hardness and roughness of the materials, indicating a complex interaction between antimicrobial agents and the mechanical properties of soft denture liners [1,12,29,37,40].

The integration of antimicrobial agents into SDLM has been scrutinized in various studies, revealing a spectrum of outcomes regarding their effects on the physical and mechanical properties of denture bases. The findings indicate a lack of consensus among the research community. Some studies reported no significant impact on mechanical characteristics upon the incorporation of antimicrobial agents, whereas others noted detrimental effects on specific mechanical properties. Notably, nystatin did not alter the tensile strength, hardness, water sorption, or solubility of soft denture liners [35,41,52]. Conversely, the inclusion of chlorhexidine was associated with a decrease in the peel bond strength of these liners [28]. The application of silver nanoparticles has been shown to enhance the hardness and water-sorption properties of denture liners [1]. Additionally, the incorporation of neem and garlic diminished the adherence of *C. albicans* without negatively impacting the hardness of the soft denture liners, while cinnamaldehyde demonstrated the potential to increase the rigidity of these materials [35,43].

In terms of methodology, the predominant cultural approach for cultivating fungal strains has involved the use of Sabouraud’s agar as the primary culture medium. Sabouraud’s broth was utilized in five studies [27,37,44,46,47], brain-heart infusion broth in three studies [28,31,43], and YEPD agar in two studies [32,41]. Catalán et al. employed Sabouraud’s agar for their in vitro research, transitioning to brain–heart infusion broth for their in vivo experiments [33]. An analysis of the literature indicates a preponderance of in vitro studies over in vivo investigations concerning the efficacy of treatments for stomatitis. However, there is a compelling need for the advancement of more sophisticated in vivo methodologies to replicate physiological conditions within the human body more accurately. The findings of this systematic review align with those of another comprehensive review, which noted significant antibacterial activity associated with the antimicrobial agents; nevertheless, their physical and mechanical properties remained largely unaffected or unchanged. Notably, increased concentrations of these agents were found to alter their optical properties [56] markedly.

A significant challenge encountered during this systematic review was the variability in study design, agent concentrations, and testing protocols across the included literature. The lack of standardization in outcome measurements—such as methods for quantifying *Candida albicans* inhibition, exposure time frames, and mechanical testing conditions—limits the comparability between studies and hinders meta-analytic synthesis. Additionally, discrepancies in the units used (e.g., wt%, ppm, μg/mL) further complicate direct efficacy and material impact comparisons. These inconsistencies highlight the pressing need for harmonized evaluation criteria in future research. Beyond the laboratory, few studies addressed the clinical implications of agent incorporation, such as mucosal compatibility, patient-reported outcomes, or esthetic stability over time. As such, future investigations should emphasize long-term in vivo performance, agent release kinetics, and toxicity evaluations, particularly for agents with sustained antifungal activity. This would bridge the current gap between promising in vitro findings and real-world clinical application.

### Limitations and Future Directions

Despite the comprehensive nature of this review, certain limitations must be acknowledged. The majority of the included studies were in vitro in design, which, while valuable for establishing baseline efficacy, do not fully replicate the complex conditions of the oral environment. Variables such as saliva composition, pH fluctuations, oral microbiota diversity, prosthesis wear, and patient hygiene behaviors are difficult to simulate in laboratory settings, thereby limiting the direct clinical applicability of the in vitro findings.

Additionally, there was substantial heterogeneity among the studies regarding the type of antimicrobial agents, soft denture liner materials used, concentration ranges, exposure times, and methods of microbial assessment. This variability not only precluded quantitative synthesis through meta-analysis but also complicated direct comparisons between studies. Differences in the units of concentration (e.g., weight percentage, parts per million, or μg/mL) and in the evaluation protocols (e.g., CFU counting, agar diffusion, SEM analysis) further constrained the ability to draw consistent conclusions.

Another limitation pertains to the limited exploration of long-term effects. Few studies examined the sustained release profiles of antimicrobial agents, changes in physical properties over time, or their impact on esthetic parameters such as color stability and surface gloss. Moreover, only a handful of investigations considered the potential cytotoxicity, biocompatibility with oral tissues, or patient-centered outcomes such as comfort, taste, or mucosal response.

Future research should prioritize high-quality in vivo studies that not only assess antifungal efficacy but also account for clinical parameters including mucosal health, prosthesis retention, and patient satisfaction. Standardized testing protocols across studies would enhance reproducibility and allow for meaningful comparisons. Moreover, investigating controlled-release delivery systems—such as nanocarriers or microparticles—could offer more consistent and prolonged antifungal activity. Research into synergistic agent combinations (e.g., herbal and synthetic) and their effects on both mechanical properties and microbial resistance is also warranted. Finally, attention should be paid to agent–material compatibility tailored to specific liner types (e.g., tissue conditioners vs. silicone-based liners) to ensure optimal clinical outcomes without compromising material integrity.

## 5. Conclusions

The incorporation of antimicrobial compounds into soft denture liner materials (SDLMs) has demonstrated significant efficacy, with tissue conditioners emerging as the preferred substrate.

- Among the agents studied, nystatin was the most frequently employed, exhibiting robust fungicidal properties in both in vivo and in vitro investigations.
- The antimicrobial agents effectively inhibited the adhesion and colonization of *Candida albicans*, thereby serving as a preventive measure against denture-induced stomatitis.
- The enhancement of antifungal effects was generally aligned with increasing concentrations of antimicrobial agents. However, several compounds, including carvacrol, *Melaleuca alternifolia*, chlorhexidine diacetate, and itraconazole, were observed to retain efficacy at comparatively lower concentrations.
- A range of other agents, such as miconazole, ketoconazole, Ag-zeolite, fluconazole, *Carum anthelminticum*, ocimum sanctum seed oils, quaternized chitosan, neem, terpinen-4-ol, and cinnamaldehyde, demonstrated complete inhibition of *C. albicans* growth across various concentrations.
- The introduction of these antimicrobial agents had a minimal impact on the mechanical properties of the soft liners, with alterations being proportional to the concentrations used.
- While the addition of antimicrobial substances to soft liners appears to influence the mechanical characteristics of the denture bases, the prevailing evidence remains inconclusive.
- Further research is warranted to elucidate the long-term implications of these modifications on the performance and durability of denture materials.

## Figures and Tables

**Table 1:** Characteristics of in vitro studies.

Study	Type SDLM	No. of Samples	Antimicrobial Agent	Concentration	Conclusions
Thomas et al., 1978 [52]	TC	6	Nystatin Amphotericin B	0, 500,000 U, 1,000,000 U 0, 10 mg, 20 mg	Nystatin completely inhibited *C. albicans* at 1,000,000 U Amphotericin very little inhibition
Quinn et al., 1985 [49]	TC	24	Miconazole Ketoconazole Amphotericin B Nystatin	250 mg 200 mg 10 mg, 20 mg 500,000 U	Amphotericin B ineffective Miconazole & ketoconazole & nystatin completely inhibited *C. albicans*
Nikawa et al., 1997 [47]	TC	96	Ag-zeolite	1, 2, 3, 4, 5%	Ag-zeolite completely inhibited *C. albicans* at 5%
Matsuura et al., 1997 [45]	TC	11	SZ	0%, 2%	SZ at 2% antimicrobial effect
Chow et al., 1999 [34]	TC	114	Nystatin, Fluconazole, Itraconazole	1%, 3%, 5%, 7%, 9%, 11%	Itraconazole 5% wt/wt most fungicidal activity
Catalán et al., 2008 [33]	TC	-	*Melaleuca* *arternifolia* Nystatin	0, 0.5, 1.0, 1.5, 2.0 mL 1.0 mL	*Μ. arternifolia* inhibited *C. albicans* at 1.0 mL Nystatin completely inhibited *C. albicans*
Nam 2011 [46]	TC	162	Silver NPs	0, 0.1, 0.5, 1.0, 2.0, 3.0%	Silver NPs antimicrobial properties at 2, 3%
Falah-Tafti et al., 2010 [36]	TC	24	Nystatin Fluconazole	1–10% 10%	Nystatin at 1% to 10% & Fluconazole 10% completely inhibited *C. albicans*
Chladek et al., 2011 [1]	SB	8	AgNPs	0, 10, 20, 40, 80, 120, 200 ppm	Highest antifungal efficacy at 200 ppm
Gonçalves et al., 2012 [39]	AB	400	Undecylenic Acid	10%	UDA ineffective
Srivatstava et al., 2013 [51]	TC	90	Origanum Oil	0, 10, 20, 30, 40, 50, 55, 57, 60, 65%	Origanum oil reduced fungal adherence and colonization of *C. albicans* at 60, 65 vol%
Bertolini et al., 2014 [31]	AB	144	CDA, CDH	0, 0.5, 1.0, 2.0 wt%	CDA reduced the biofilm development of *C. albicans* at 2% CDH ineffective
Bueno et al., 2015 [32]	TC& AB	1488	Nystatin, Ketoconazole Miconazole, Itraconazole Chlorhexidine diacetate	0.016, 0.032, 0.064, 0.128 … plus 0.256 … plus 0.384 g/mL	Nystatin at 0.064 & CDA at 0.064 g/mL completely inhibited *C. albicans* Ketoconazole at 0.128 Miconazole& Itraconazole at 0.256 maximum antifungal effect
Muttagi et al., 2017 [8]	TC	200	*C. Anthelminticum*, *O. Sanctum* Linum usitatissimum	600, 700, 800 μL 800, 900, 1000 μL	*C. Anthelmintic* & *O. Sanctum* completely inhibited *C. albicans* at 800 μL Linum usitatissimum ineffective
Vankadara et al., 2017 [53]	TC	160	*Melaleuca alternifolia*	10, 20, 30, 40%/0.5, 1, 1.5, 2 mL	*Μ. arternifolia* antifungal efficacy at 40%
Baygar et al., 2018 [30]	SB	-	Carvacrol	0, 0.5, 1, 2.5, 5, 10, 20, 50 μL	Carvacrol decreased (98.03%) the biofilm formation at 10 μL
Albrecht et al., 2018 [28]	AB	6	CDA	0, 1%	CDA antifungal activity at 1%
Altinci et al., 2018 [9]	SB & AB	40	BAC	0.5, 1, 2 and 5 wt%.	BAC completely inhibited *C. albicans* at 0.5, 1, 2, 5 wt%
Kim et al., 2018 [41]	TC	5	Nystatin-alginate MPs	2 *w*/*v* nystatin mixed with 0.5% alginate	MPs demonstrated antifungal activity at 2 mg
Kumar et al., 2018 [43]	AB	30	Garlic (Allium sativum) Neem (*A. indica*)	50, 100, 200, 400, 500 μg	Neem completely &Garlic partially inhibited *C. albicans*. The most efficient concentration was not mentioned
Lee et al., 2018 [44]	TC	216	CS QCS	0, 5, 7.5, 10%	CS showed greater antifungal activity at 7.5% QCS completely inhibited *C. albicans* at 7.5 & 10%
Maior et al., 2019 [35]	TC	24	Terpinen-4-ol Cinnamaldehyde	0.125, 0.25, 0.5, 1, 5, 10, 20, 30, and 40% ...plus 0.0156%	Cinnamaldehyde completely inhibited *C. albicans* at 20, 30, 40% T-4-ol presented antifungal effect but even in concentration 40% were still viable *C. albicans* cells
Kreve et al., 2019 [42]	AB	100	AgVO3	0, 1, 2.5, 5, 10%	AgVO3 was most efficient against *C. albicans* at 5%
Ansarifard et al., 2021 [29]	AB	80	CuO NPs	0, 0.5, 5, 50, 500 µg/mL	NPs significantly inhibited (75%) *C. albicans* at 500 μg/mL
Deng et al., 2021 [12]	AB	10	AgNPs	0, 0.1, 0.2, 0.3%	AgNPs significantly inhibited (63.38%) & (75.51%) *C. albicans* at 0.2 & 0.3% respectively
Habibzadeh et al., 2021 [40]	SB	20	AgNPs	0, 0.5, 1.0, 2.0, 3.0 wt%	greatest antifungal efficacy at 3 wt% AgNPs
Lee et al., 2021 [23]	TC	-	CO	0, 200, 400, 600 μg/mL	CO antimictobial efficacy at 600 μg/mL
Songsang et al., 2022 [50]	TC	25	Litsea cubeba	0, 5, 10, 20, 30% *v*/*v*	Litsea cubeba significantly inhibited *C. albicans* in 10, 20, 30% *v*/*v*
Ferreira et al., 2022 [37]	TC	72	SZZ-NPs Fluconazole	0, 0.5, 2% *w*/*w* 5% *w*/*w*	Fluconazole short-term inhibitory effect at 5% *w*/*w* and SZZ-NPs long-term inhibitory effect at 2% *w*/*w*
Ahmed et al., 2023 [27]	AB	40	Titanium Oxide NPs	0, 1.0, 1.5, 2.0 wt%	Antifungal effect: 1.0% -> 91.42% 1.5% -> 95.57% 2.0% -> 99.28%

AgNPs, silver nanoparticles; BAC, Benzalkonium Chloride; CDA, Chlorexidine diacetate; CDH, Chlorexidine hydrochloride; CS, Chitosan; CO, Cnidium Officinale; QCS, Quaternized chitosan poly grafted; SZ, Silver-zeolite; SZZ NPs, Silver zinc zeolite nanoparticles.

**Table 2:** Characteristics of in vivo studies.

Study	Type SDLM	No. Samples	Antimicrobial Agent	Concentration	Conclusions
Catalán et al., 2008 [33]	TC	27	*Melaleuca* *arternifolia* Nystatin	-	Both antimicrobial agents significantly inhibited *C. albicans*
Geerts et al., 2008 [38]	TC	40	Νystatin	500,000 U	Nystatin completely inhibited *C. albicans* at 500,000 U
Saravanan et al., 2015 [7]	AB	30	Silver—Zeolite	0, 5%	Silver zeolite significant antimicrobial effect at 5%
Procópio et al., 2022 [48]	AB	40	Nystatin Chlorexidine Diacetate	0.032 g 0.064 g	Interim resilient liner modified by nystatin and chlorhexidine at MICs for *C. albicans* biofilm is a viable optional approach for DIS treatment

**Table 3:** Assessment of risk of bias using ROBINS tool for non-randomized studies.

Study	Pre Intervention		At Intervention		Post Intervention			Overall RoB
	Bias Due to Confounding	Bias in Selection of Participants	Bias in Classification of Interventions	Bias Due to Deviations from the Interventions	Bias Due to Missing Data	Bias in Measurements of Outcomes	Bias in Selection of Reported Results	Low/Moderate/Serious
Ahmed 2023 [27]	Low	Moderate	Low	Low	?	Low	Low	Moderate
Albrecht 2018 [28]	Low	Low	Low	Low	?	Low	Low	Low
Altinci 2018 [9]	Low	Moderate	Moderate	Low	?	Low	Low	Moderate
Ansarifard 2021 [29]	Low	Low	Low	Low	?	Low	Low	Low
Baygar 2018 [30]	Low	Moderate	Low	Low	?	Moderate	Low	Moderate
Bertolini 2014 [31]	Low	Low	Low	Low	?	Low	Low	Low
Bueno 2015 [32]	Low	Low	Low	Low	?	Low	Low	Low
Catalán 2008 [33]	Low	Low	Low	Low	?	Low	Low	Low
Chladek 2011 [1]	Low	Moderate	Low	Moderate	?	Low	Low	Moderate
Chow 1999 [34]	Low	Low	Moderate	Low	?	Low	Low	Moderate
Deng 2021 [12]	Low	Moderate	Low	Low	?	Low	Low	Moderate
Falah-Tafti 2010 [36]	Low	Moderate	Low	Low	?	Low	Low	Moderate
Ferreira 2022 [37]	Low	Moderate	Low	Low	?	Low	Low	Moderate
Geerts 2008 [38]	Low	Low	Low	Low	?	Low	Low	Low
Habibzadeh 2021 [40]	Low	Low	Low	Low	?	Low	Low	Low
Kim 2018 [41]	Low	Low	Low	Low	?	Low	Low	Low
Kreve 2019 [42]	Low	Moderate	Low	Moderate	?	Low	Low	Moderate
Kumar 2018 [43]	Low	Low	Moderate	Low	?	Low	Low	Moderate
Lee 2018 [44]	Low	Low	Low	Low	?	Low	Low	Low
Lee 2021 [23]	Low	Low	Low	Low	?	Low	Low	Low
de Fátima Souto Maior 2019 [35]	Low	Moderate	Low	Low	?	Low	Low	Moderate
Matsuura 1997 [45]	Moderate	Low	Moderate	Low	?	Low	Low	Moderate
Muttagi 2017 [8]	Low	Moderate	Low	Low	?	Low	Low	Moderate
Nam 2011 [46]	Low	Low	Low	Low	?	Low	Low	Low
Nikawa 1997 [47]	Low	Moderate	Moderate	Low	?	Low	Low	Moderate
Quinn 1985 [49]	Low	Moderate	Low	Moderate	?	Low	Moderate	Moderate
Saravanan 2015 [7]	Moderate	Low	Low	Low	?	Moderate	Low	Moderate
Songsang 2022 [50]	Low	Low	Low	Low	?	Low	Low	Low
Srivatstava 2013 [51]	Low	Low	Low	Low	?	Low	Low	Low
Thomas 1978 [52]	Moderate	Moderate	Low	Low	?	Low	Moderate	Moderate
Vankadara 2017 [53]	Moderate	Low	Low	Low	?	Moderate	Moderate	Moderate

The question mark (“?”) symbol indicates an unclear risk of bias in areas where the primary study did not provide enough information for an objective assessment.

**Table 4:** Assessment of risk of bias using ROB2 tool for randomized controlled trials.

Authors	Bias from Randomization Process	Bias Due to Deviation from Intervention	Bias Due to Missing Outcome Data	Bias in Measurement of Outcome	Bias in Selection of Reported Results	Overall Bias
Gonçalves 2012 [39]	No	No	No	No	Yes	No
Procópio 2022 [48]	No	No	Some concerns	Yes	No	Some concerns

**Table 5:** Synthesized Overview of Antimicrobial Agents Incorporated into Soft Denture Liners.

Antimicrobial Agent	Number of Studies	Effective Concentration	In Vitro/In Vivo	Impact on SDLM Properties
Nystatin	8	500,000–1,000,000 U	Both	Minimal
Chlorhexidine	5	0.064–1%	Both	Possible peel strength reduction
AgNPs	6+	0.2–3%, 200 ppm	Mostly in vitro	↑ Hardness, water sorption
Silver-Zeolite	2	5%	In vitro & in vivo	None
Miconazole	3	0.128–0.256 g/mL	Both	↑ Hardness
Ketoconazole	2	200 mg	In vitro	None
Fluconazole	2	10%	In vitro	None
Itraconazole	2	0.032–0.256 g/mL	In vitro	↑ Hardness
BAC	1	0.5–5 wt%	Both	None
Neem	2	200–500 μg	In vitro	None
Garlic	2	50–400 μg	In vitro	None
Cinnamaldehyde	2	20–40%	In vitro	↑ Rigidity
Carvacrol	1	10 μL	In vitro	None
Terpinen-4-ol	1	≤40%	In vitro	↑ Hardness

↑ means increase.

## Data Availability

The original contributions presented in this study are included in the article. Further inquiries can be directed to the corresponding author.
